# A Gold Nanoparticle and Aflatoxin B1-BSA Conjugates Based Lateral Flow Assay Method for the Analysis of Aflatoxin B1

**DOI:** 10.3390/ma5040634

**Published:** 2012-04-13

**Authors:** Jihea Moon, Giyoung Kim, Sangdae Lee

**Affiliations:** Post-Harvest & Food Engineering Division, Department of Agricultural Engineering, National Academy of Agricultural Sciences, Suwon 441-707, Korea; E-Mails: mmir95@gmail.com (J.M.); sdlee96@gmail.com (S.L.)

**Keywords:** aflatoxin B1, gold nanoparticle, immune-chromatographic assay, dot strip assay, mycotoxin detection

## Abstract

A rapid and simple immuno-chromatographic assay was developed to detect aflatoxin B1 (AFB1). The assay was based on a modified competitive binding format using colloidal gold and polyclonal antibody (Pab) conjugates. The anti-AFB1 Pab was immobilized to a defined detection zone on a porous nitrocellulose membrane and colloidal gold particles were conjugated to AFB1-BSA which served as a detection reagent. The AFB1-containing sample was added to the membrane and allowed to move with AFB1-BSA-coated particles dried on the conjugation pad. The mixture was then passed along the porous membrane by capillary action past the Pab in the detection zone, which captured AFB1 or AFB1-BSA. AFB1 in the sample inhibits binding of AFB1-BSA conjugated gold particles to the Pab and prevents formation of a red color dot. In the absence of AFB1, AFB1-BSA conjugated gold particles bound to the Pab, give a red color within this detection zone. With this method, 10 μg/mL of AFB1 was detected in less than 10 min. The developed AFB1 assay also showed no cross reaction to Ochratoxin A (OTA).

## 1. Introduction

Aflatoxin B1 (AFB1), produced by *Aspergillus flavus* and *A. parasiticus*, is one of the most poisonous toxins in the world. Exposure to this toxin induces higher risk of the incidence of hepatocellular carcinomas [1]. The international Agency for Research on Cancer (IARC) has classified the AFB1 as Group 1 carcinogen [2]. AFB1 could be produced in a number of agricultural products under favorable growth conditions (temperature, humidity, and pressure) during storage [3]. AFB1 is known to occur naturally in agricultural products such as peanuts, corn, and animal feeds [4]. These contaminated food or feeds present serious health hazards [1]. Also, aflatoxin B is toxic and carcinogenic in animals and many countries have set regulations for human food and animal feeds. In the European Union, the maximum residue levels (MRLs) are 2 μg/kg in nuts and dried fruits, and 4 μg/kg in cereals. In Korea, MLs are 10 μg/kg and 50 μg/kg in human food and animal feed [5], respectively. Because AFB1 is heat stable, the initial contamination levels could persist to the final product and finally reach the human or animal digestive tract. Therefore, to protect human and animal health, early prevention of contaminations of grains and animal feeds from this toxin is necessary.

Current analytical procedures are based on the time-delayed analysis of AFB1 by thin-layer chromatography (TLC), high-performance liquid chromatography (HPLC), over pressured-layer chromatography (OPLC), and enzyme-linked immunosorbent assay (ELISA) [6]. These measurements have to be performed within a laboratory and require one to several hours, although they have excellent detection limits ranging from 1~6 ng/L [6,7]. To reduce the contamination of AFB1 during distribution and storage of grains and animal feeds, a rapid and easy-to-use detection method is required.

One of the quick on-site assessment methods for AFB1 contaminations is an immunochromatographic lateral flow dipstick assay [8]. The lateral flow assay is an easy and fast detection method that is very reliable and cheap in production. Due to their reliability and rapidity, strip assays are well known in the medical field for diagnosing blood infections. drug use, or for detecting pregnancy [9].

Construction of a lateral flow strip comprises four different pads; the sample application pad, the conjugate release pad, the nitrocellulose membrane, and the absorption pad. The assay is based on the retention and visual detection of color-labeled antibodies during their flow through two lines in a membrane. When running the assay, the sample is driven up the strip by capillary forces where it is focused in capture lines corresponding to immobilized immunoreagents [9]. It is then, flow reacted with immobilized antigen to generate signals.

Recently, the strip assay has been applied to develop portable kits for on-site monitoring of AFB1 in grains [6,10,11]. In particular, some strip assays for the qualitative and semi-quantitative detection of AFB1 in food and feed have been described [5]. These competitive strip assay systems for AFB1 detection were based on colloidal gold-antibody conjugates.

Colloidal gold-antibody conjugates are widely used labeling substance to detect antigens. However, the antibody is too sensitive to maintain an active binding state when other components are attached and might lose colloidal stability [12]. Bovine serum albumin (BSA) has more structural stability than an antibody. Accordingly, colloidal gold-BSA conjugates have been used as a strategy for increasing colloidal stability and enhancing biocompatibility in various nanoparticle systems [13]. Therefore, if colloidal gold is conjugated with AFB1-BSA instead of AFB1-Pab, it could enhance stability, sensitivity, and selectivity of strip assay system.

The goal of this study was to develop a more stable, rapid and simple detection method than the conventional strip method. The one dot strip assay was developed based on lateral flow assay, and applied to modify one-step membrane assay. Preparation and characterization of colloidal gold-antigen (AFB1-BSA) conjugates and their use in developing a competitive immunoassay for on-site assessment of AFB1 was studied. A one-dot strip assay which can preliminarily analyze AFB1 levels has been developed.

## 2. Results and Discussion

Established strip assays for AFB1 analysis are composed of a test line and a control line in the membrane. By comparing the readings of strips the results can supposedly be presented. However, they depend on the concentration of the color markings of analysis to develop ways to streamline [9]. Established strips were considered for adaptation to electronic readers for comparing evaluation between test line and control line for improving the sensitivity of detecting AFB1 in samples [10]. Other strip assays have been developed for identification with naked eye, but shortcomings have been pointed out about variability from strip to strip due to the quantity of gold-antibody conjugates. Therefore, there has been research on other methods aimed at minimizing handling and evaluating signal differences to compliment their shortcomings [9].

When BSA, a capping agent for nanoparticles, was conjugated with gold nanoparticles, its bioconjugate nanoparticles were chemically changed. These conjugates could enhance colloidal stability, and biochemical activity [13]. In this study, this specificity of BSA was used to improve the sensitivity and detection time of strip assay. Also, another purpose of the study was to simplify the manufacturing process and detection reading of strip assays. Therefore, in this study, AFB1-BSA-gold conjugates were used as competitive binding molecules for the improvement of efficacy and stability of detection.

### 2.1. Interpretation of Test Results

**Figure 1:**
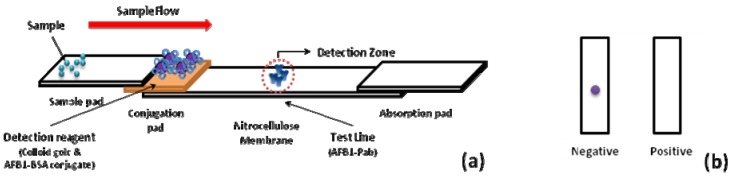
(**a**) The schematic description of the immunochromatographic test device. (**b**) Illustrations of immunochromatographic test results. One-dot strip assay was composed of a conjugation pad, membrane, sample pad, and absorption pad. The detection is based on the competition of aflatoxin B1 (AFB1) in a sample and AFB1-BSA-gold nanoparticles in binding to the antibody of AFB1 immobilized on a membrane. (Bovine serum albumin = BSA).

A schematic description of the immunochromatographic test device is shown in Figure 1a. In the detection zone, the AFB1-Pab was immobilized onto the test region. A positive result was judged by the disappearance of one dot in the membrane. This was due to the fact that AFB1 in the sample bound to AFB1-Pab in the membrane, and thus AFB1-BSA coated colloidal gold particles could not bind to Pab in the test region. However, in the blank test, AFB1-BSA-gold conjugates which reacted with AFB1-Pab, could be trapped in the test region. Therefore, a negative result was judged by the appearance of a single dot in the test strip (Figure 1b).

### 2.2. Optimal Condition Studies for Preparation of AFB1-BSA-Gold Nanoparticles

In this experiment, colloidal gold nanoparticles are the most important part of the test strip. Because the detection by using immuno-gold has specificities: colloidal gold is formed in solution by the balance between electrostatic repulsion and Van der Waals attraction among the particles [12]. However, these forces change greater than the counteraction on addition of ionic substances. It leads to an aggregation accompanying a color change from red to blue or gray [14]. Also, colloidal gold has good mobility in the porous membrane, and low susceptibility to aggregation during the preparation of the test strip. According to these advantages, gold nanoparticles provide the opportunity to improve the assay sensitivity and the simplicity of a test strip [15]. Optimal conditions of pH and AFB1-BSA concentration for conjugation can be determined by comparing the adsorption at 520 nm and the color shown.

#### 2.2.1. Effect of pH

To study the effect of pH, solutions containing gold nanoparticles were adjusted to pH 5.4–10.1. The same volume of NaCl solution (1.0 M) was then added and the color of the obtained solutions was observed after 10 min. Because high salt concentrations induce gold nanoparticle aggregation, an insufficient amount of antigens are adsorbed on the surface of the nanoparticles. At this point, the aggregation could be visually detected changing from red color to blue-grey [9].

**Figure 2:**
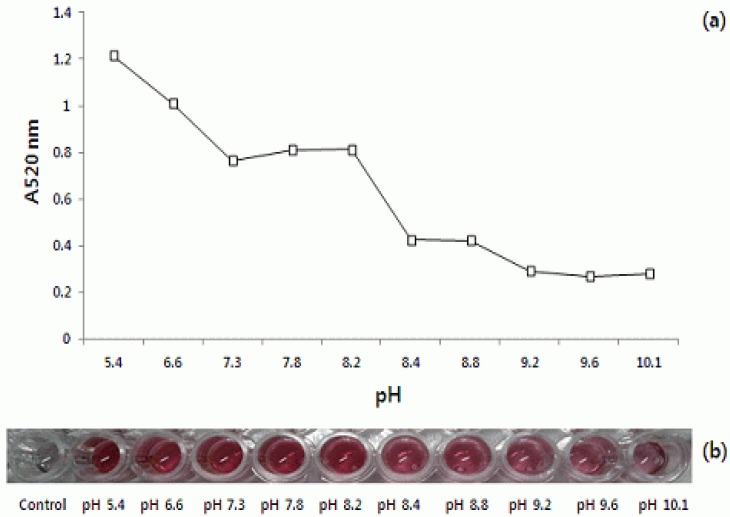
(**a**) Optimization of experimental conditions for preparation of colloidal gold-BSA conjugates; (**b**) Visual influence of pH solution on the degree of conjugation of colloidal gold solutions.

Figure 2a shows the absorbance of mixed solutions at 520 nm. In Figure 2b, color shown, the optimal conditions of the pH for the conjugation were determined. For the variation of pH, a maximum value of 520 nm was reached at pH 5.4, but no significant change occurred from pH 7.3 to 8.2 (Figure 2a). However, when the pH was above 8.2, a color change from red to red-purple was seen. A purple color of nanoparticles could be interpreted as being aggregated [16]. Therefore, pH 7.3 of colloidal gold was considered to be suitable to conjugate with AFB1-BSA. Because the pH of buffer and blocking solutions was 7.4, the colloidal gold solution was adjusted to pH 7.3 by considering the optimal binding activities of AFB1-BSA-gold conjugate. This meant that Au conjugates and NaCl could not be aggregated.

#### 2.2.2. Effect of AFB1-BSA Concentrations

**Figure 3:**
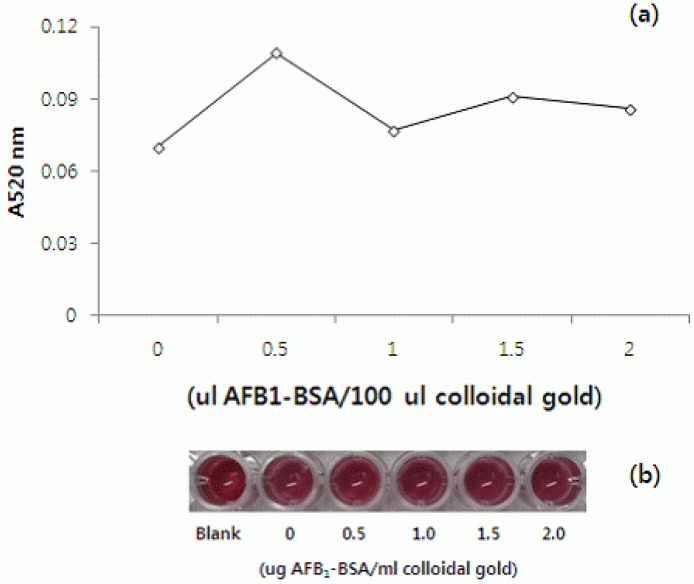
(**a**) Optimization of experimental conditions for preparation of colloidal gold-BSA conjugates; (**b**) Visual effect of the AFB1-BSA concentration on the conjugation yields, under optimal pH conditions.

The optimal AFB1-BSA concentration conjugating with colloidal gold had to be determined. The reference experiment was carried out with various amounts (0–2.0 μL) of AFB1-BSA (2.0 mg/mL in PBS) after adding 100 μL colloidal gold adjusted to the optimal pH 7.3. The decrease in absorbance values showed that the efficacy of coating AFB1-BSA on the surface of colloidal gold particles was reduced [12]. In this study, similar results showed that 0.5 to 2.0 μL AFB1-BSA were stabilizing in 100 μL colloidal gold (Figure 3a,b). Firstly, 0.5 μg AFB1-BSA was chosen, to be evaluated as the maximum value at 520 nm and the minimum content for stabilization of colloidal gold. However, it was too small to detect AFB1 in the one dot assay, because AFB1-BSA-gold nanoparticles could not bind to AFB-Pab on the membrane (data not shown). Therefore, 1.0 μg AFB1-BSA was chosen for use according to the detecting efficacy of AFB1 on a one dot strip assay.

### 2.3. Detection Limit of AFB1 Test Strip

The hypotheses of this study are as follows: (1) The molecular weight of AFB1 is lower than that of AFB1-BSA-gold conjugates, and the moving velocity of AFB1 in the sample is higher than for AFB-BSA conjugates; (2) AFB1-PAb is only able to combine with AFB1 or AFB1-BSA; (3) If there is AFB1 in the samples, AFB1 is faster combined with AFB1-PAb on the membrane than the AFB1-BSA-gold particle conjugates. At this point, the inhibition assay is completed and color detection does not occur. However, if there is no AFB1 in the sample, AFB1-BSA-gold particle conjugates combine with AFB1-Pab and can be detected with the naked eye with red coloration.

**Figure 4:**
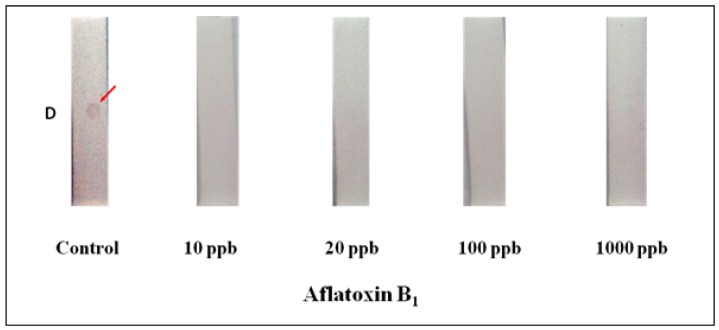
Immunochromatographic detection of AFB1. A series of dilutions (10–1000 ng/mL) of AFB1 was prepared in phosphate buffered saline (PBS) containing 10% methanol. Details of the preparation of the lateral flow test device and assay are described in the text. Binding inhibitions are shown in the presence of AFB1. “D” is the detection zone of the membrane.

In order to evaluate the feasibility of dot-strip assay, we studied quantitative detection of AFB1 by using a test strip. AFB1-PAb served as the probe, colloidal gold and AFB1-BSA conjugates acted as the coating antigen and AFB1 acted as the competitive antigen. AFB1 and Au-AFB1-BSA conjugates compete for the limited AFB1-PAb binding sites. With a concentration of the coating antigen of 0.1 μL/mL of colloidal gold, AFB1-PAb conjugates were diluted 10,000 fold into PBS buffer, the sensitivity for AFB1 detection was below 10 ng/mL by visual observation (Figure 4). There were similar detection limits with other developed strip assays for the detection of AFB1 [5,8,9,10,12]. Also, this detection limit was comparable to those obtained using other strip assays (below 10 ng/mL for AFB1 and ochratoxin A in chili), including NRL array biosensors (1.0 ng/mL in buffer), electrochemical immunosensors (below 12 ng/mL in buffer), and micro-comb electrodes (below 10 ng/mL in buffer) [3,11,17,18]. Analysis was complete in ten minutes. In the absence of AFB1 (PBS only), AFB1-BSA-gold conjugates were bound to AFB1-PAb, while inhibition binding was detected in the presence of AFB1. Non-specific binding of reagents was very low. However, further research would be required to improve the sensitivity for the analysis in real samples.

### 2.4. Cross Reactivity of AFB1 One-Dot Test Strip

**Figure 5:**
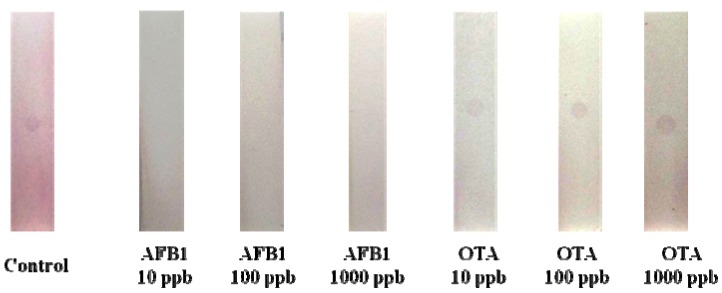
Cross reactivity of AFB1 test strip. The Ochratoxin A (OTA) test solution was analyzed by AFB1 test strip. Details of the preparation of the lateral flow test device and assay are described in the text. Binding inhibition is shown in the presence of AFB1.

This method had good specificities for Ochratoxin A (OTA), OTA is a toxin, similar to AFB1, mainly produced by *Aspergillus* spp. Also, OTA and AFB1 are very hazardous to grains and animal feeds [1]. Therefore, the cross reactivity of the AFB1 test strip with OTA was examined. When 10, 100, and 1000 ng/mL of OTA was tested, specific binding was not revealed in the test strip (Figure 5). It was thus shown that the one-dot test strip certainly can be used widely for the determination of AFB1 in animal feeds or food.

## 3. Experimental Section

Aflatoxin B1 (AFB1), Ochratoxin A (OTA), aflatoxin B1-bovine serum albumin (AFB1-BSA) conjugate, anti-mouse IgG, polyclonal antibody (PAb) to AFB1, were purchased from Sigma (ST. Louis, MO, USA). The Hi-Flow Plus 240 membrane cards, Glass Fiber conjugate pads, and Cellulose Fiber sample pads were obtained from Millipore (Bedford, MA, USA). Nanocolloidal gold particles (diameter 40 nm) were purchased form BioAssay Works (Ijamsville, MD, USA).

AFB1-BSA was conjugated to colloidal gold particles, under variable conditions of pH and AFB1-BSA concentration, to determine the optimal conditions. The pH of the colloidal gold suspension was adjusted in the range 5.4–10.1 by adding different buffer solutions (BioAssay Works. MD, USA). AFB1-BSA solution was diluted to 2.0 mg/mL solution of 0.05 mM phosphate buffered saline (PBS, pH 7.2). Then 1.0 μL of AFB1-BSA diluted solution was mixed with 100 μL colloidal gold solution at various pHs in wells of 96-well microplates. The mixtures were shaken 2–3 s and then incubated for 30 min at room temperature. Ten μL of Blocker BSA solution was added for blocking of the residual surface of the nanocolloidal gold particles. The pH optimum was noted of the final solution. One hundred μL of NaCl solution (1.0 M) and gold binding solution were mixed into each well. Then, the mixed solutions were left for an additional 10 min to react at room temperature. These solutions were monitored by absorbance at 520 nm [12]. The pH optimum was noted from the color change of the solution. When an insufficient amount of antigens have been adsorbed on the surface of the nanoparticles, high salt concentrations induce gold nanoparticle aggregation. This aggregation can be detected because of a change in the gold colloid color from red to blue or purple. The mixture, for detecting red color at the optimal pH condition, was stored at 4 °C before use.

A picture was constructed of colloidal gold-labeled AFB1-BSA, to determine the optimum amount of protein needed to coat the surface of the gold particles. Various volumes (0, 0.5, 1.0, 1.5, 2.0 μL) of AFB1-BSA solutions (2.0 mg/mL) were added to each well in 96 well microplates containing 100 μL colloidal gold adjusted to the optimal pH (7.3). The solutions were left to react for 30 min at room temperature, then 100 μL of 10% NaCl was added to each well and the solutions left for an additional 10 min at room temperature. If the color of the mixture did not change from red to purple or gray within 10 min, it showed that the amount of AFB1-BSA was sufficient. In this way, the necessary amounts of AFB1-BSA and colloidal gold solution were calculated.

The sample pad was prepared according to Lai [19]. It was treated with 50 mM borate buffer, pH 7.4, containing 1% BSA and 0.05% Tween-20, and then dried overnight at 37 °C. The nitrocellulose membrane and conjugation pad were prepared according to previous studies [20]. The nitrocellulose membrane was immersed with PBS buffer. The conjugation pad was treated with 50 mM borate buffer, pH 7.4, containing 10% sucrose, 2% BSA and 0.05% Tween-20. And then, the membrane and pad were dried to the same stage as the sample pad. The test spot on the nitrocellulose membrane was formed with 1.0 μL of AFB1-PAb (2.0 mg/mL in PBS), and allowed to dry at 37 °C for 3 h. The colloidal gold-AFB1-BSA (1.0 μL/strip) was applied to a conjugate pad and completely dried at 37 °C for 3 h. The sample pad, conjugate pad, nitrocellulose membrane, and absorption pad were assembled as the lateral flow strip (Figure 1).

Detection of AFB1 with an immunochromatography strip was evaluated by measurements of the standards which were prepared by diluting a stock solution with methanol: PBS (1:9, v/v). In the assay, 100 μL of standard solution was added into the sample-well of the strip device. The competitive reaction between the conjugate and analytes took place immediately and carried through to the next membrane with the immobilized binder. The analytes or gold mixtures competitively bind with AFB1-PAb in the membrane. As soon as all the solution reached the end of the strip, usually within 10 min, the color dots were produced in the strip. For the cross reactivity of the test strip, samples containing 10, 100, and 1000 μg/mL of OTA were also assayed by the AFB1 test strip to evaluate cross reactivity.

## 4. Conclusions

The one dot strip assay in this study is able to decrypt with the presence or absence of a dot. The specificity of this assay is to simplify the processing and decoding steps of established strip assays, as follows: The processing stage for control lines is removed. Firstly, applicability is improved in developing multi strip assays for detection of multiple mycotoxins. Secondly, manufacturing costs would be reduced.

In this study, only AFB1-PAb was used to probe on the strip. Other antibodies will be used with multiple probes in further study. It may be possible to develop multiple detection of mycotoxins. The structures of established multi-strip assays were composed of the control line before and after multiple test lines of each mycotoxin. However, these dot assays are composed of test dots per mycotoxin without a control line. It means that strip assay could be used to present a variety of detection possibilities for mycotoxin, and considered more appropriate than design changes in saving the place of the control line. For development of multi dots assay, there would be a need to study the optimal concentrations of toxin and antibody, and optimal binding conditions between gold nanoparticles and mycotoxin-BSA conjugates.
